# Combination of Metabolomic and Proteomic Analysis Revealed Different Features among *Lactobacillus delbrueckii* Subspecies *bulgaricus* and *lactis* Strains While *In Vivo* Testing in the Model Organism *Caenorhabditis elegans* Highlighted Probiotic Properties

**DOI:** 10.3389/fmicb.2017.01206

**Published:** 2017-06-28

**Authors:** Elena Zanni, Emily Schifano, Sara Motta, Fabio Sciubba, Claudio Palleschi, Pierluigi Mauri, Giuditta Perozzi, Daniela Uccelletti, Chiara Devirgiliis, Alfredo Miccheli

**Affiliations:** ^1^Department of Biology and Biotechnology “C. Darwin”, Sapienza University of RomeRome, Italy; ^2^Institute of Biomedical Technologies, National Research CouncilMilan, Italy; ^3^Department of Chemistry, Sapienza University of RomeRome, Italy; ^4^Food and Nutrition Research Center, Council for Agricultural Research and EconomicsRome, Italy

**Keywords:** *Lactobacillus delbrueckii* metabolism, bacterial folate biosynthesis, bacterial galactose metabolism, tagatose pathway, lactic acid bacteria, foodborne bacteria

## Abstract

*Lactobacillus delbrueckii* represents a technologically relevant member of lactic acid bacteria, since the two subspecies *bulgaricus* and *lactis* are widely associated with fermented dairy products. In the present work, we report the characterization of two commercial strains belonging to *L. delbrueckii* subspecies *bulgaricus*, *lactis* and a novel strain previously isolated from a traditional fermented fresh cheese. A phenomic approach was performed by combining metabolomic and proteomic analysis of the three strains, which were subsequently supplemented as food source to the model organism *Caenorhabditis elegans*, with the final aim to evaluate their possible probiotic effects. Restriction analysis of 16S ribosomal DNA revealed that the novel foodborne strain belonged to *L. delbrueckii* subspecies *lactis*. Proteomic and metabolomic approaches showed differences in folate, aminoacid and sugar metabolic pathways among the three strains. Moreover, evaluation of *C. elegans* lifespan, larval development, brood size, and bacterial colonization capacity demonstrated that *L. delbrueckii* subsp. *bulgaricus* diet exerted beneficial effects on nematodes. On the other hand, both *L. delbrueckii* subsp. *lactis* strains affected lifespan and larval development. We have characterized three strains belonging to *L. delbrueckii* subspecies *bulgaricus* and *lactis* highlighting their divergent origin. In particular, the two closely related isolates *L. delbrueckii* subspecies *lactis* display different galactose metabolic capabilities. Moreover, the *L. delbrueckii* subspecies *bulgaricus* strain demonstrated potential probiotic features. Combination of *omic* platforms coupled with *in vivo* screening in the simple model organism *C. elegans* is a powerful tool to characterize industrially relevant bacterial isolates.

## Introduction

The species *Lactobacillus delbrueckii* comprises three main subspecies, namely *lactis*, *bulgaricus* and *delbrueckii*, classified in 1983 on the basis of metabolic and phenotypic properties (Weiss et al., 1983). *L. delbrueckii* subsp. *bulgaricus* and *L. delbrueckii* subsp. *lactis* are usually associated with dairy products, such as yogurt or cheeses, while *L. delbrueckii* subsp. *delbrueckii* is typical of fermented vegetables. More recently, a novel subspecies, namely *indicus*, has been described for *L. delbrueckii* isolated from Indian dairy products (Dellaglio et al., 2005). *Lactobacillus delbrueckii* is a member of the acidophilus complex, a heterogeneous group of lactobacilli related to *L. acidophilus*, comprising several bacterial species, many of which of gastrointestinal origin (i.e., *L. acidophilus*, *L. johnsonii*, and *L. gasseri*), and characterized by potential probiotic properties, particularly related to immune modulation. On the other hand, the species *L. delbrueckii* is rather known for its dairy applications in yogurt and cheesemaking (El Kafsi et al., 2014), and a deeper understanding of the mechanisms underlying its beneficial effects on human health represents a challenging goal. Traditional fermented dairy products are obtained by manufacturing procedures that often employ raw material, relying on the natural microflora preexisting in such ingredients, whose species composition reflects local environments, therefore characterized by a high biodiversity in terms of numbers of strains, whose origin is prevalently environmental (Morea et al., 1999; Tamang et al., 2016).

Several food-associated *Lactobacillus* species are also found as normal inhabitants of the human gut microbiota (Liu, 2003). The interplay between food and gut microbial consortia can affect human health status, since several foodborne strains also display probiotic features (Devirgiliis et al., 2011). Characterization of novel foodborne isolates of environmental origin is therefore crucial to predict their potential beneficial effects on human health. A broad range of different “omics” technologies are now available to allow a deep characterization of microbial physiology (Calvani et al., 2014). In particular, metabolomics and proteomics are emerging as powerful tools in the field of probiotics (Rebollar et al., 2016; Ruiz et al., 2016).

Evaluation of probiotic properties requires, along with molecular techniques, suitable *in vivo* models. The nematode *Caenorhabditis elegans* represents a valuable tool to test the effects of ingested bacteria on host physiology. *C. elegans* is a differentiated multicellular organism with a nervous system, reproductive organs, and digestive apparatus. Furthermore, it has a simple structure and a short life cycle (less than 3 days). Dietary sources, such as bacteria, play an important role in the control of *C. elegans* lifespan (So et al., 2011). A recent review elegantly discusses an important aspect concerning the relationship between bacteria and *C. elegans*: bacterial biomass indeed represents the worm food, and in this sense their trophic relationship is different from the synergistic one between mammals and gut microbiota. However, since live bacteria can influence the nematode physiology through their metabolites, the bacterial component of the *C. elegans* model can represent both direct and indirect aspects of a diet (Yilmaz and Walhout, 2014).

The nematode has been employed as a useful host for a wide variety of microbes relevant for human health (Clark and Hodgkin, 2014), including foodborne bacteria. It was recently reported that lactic acid bacteria (LAB) can exert protective functions on *C. elegans* by acting on intestinal permeability (Zhao et al., 2015). Foodborne strains of *Weissella koreensis* and *Weissella cibaria* significantly extend the lifespan of *C. elegans* (Lee et al., 2015). Moreover, in the recent past a growing body of literature demonstrated that the nematode can be successfully used to screen the probiotic features of several bacterial strains. Park et al. (2015) analyzed potentially probiotic *Bacillus licheniformis* strains isolated from traditional Korean food sources in terms of protection against *Staphylococcus aureus* infection (Yun et al., 2014), as well as of ability to enhance longevity of nematodes. Recently, *Lactobacillus gasseri* SBT2055, which had been previously demonstrated to exert beneficial effects in mice and humans, in terms of improvement of the intestinal environment and prevention of infection by influenza A virus (Nakayama et al., 2014), showed a positive impact on longevity and/or aging in the nematode through the modulation of *skn-1* gene (Nakagawa et al., 2016).

In the present work we have applied comprehensive analytical approaches based on the combination of metabolomics and proteomics as well as *in vivo* screening to compare different strains belonging to *L. delbrueckii bulgaricus* and *lactis* subspecies, in order to characterize their metabolic activities and to investigate on their potential probiotic properties.

## Materials and Methods

### Bacterial Strains and Growth Conditions

*Lactobacillus delbrueckii* subspecies used in this study were *L. delbrueckii bulgaricus* ATCC11842, *L. delbrueckii lactis* LMG6401 and the isolate *L. delbrueckii* 23 originating from Mozzarella di Bufala Campana (MBC) (Zanni et al., 2015). *Lactobacillus rhamnosus* GG (LGG, ATCC53103) was also used. All the strains were routinely maintained in Elliker broth (DIFCO) and grown at 37°C overnight under anaerobic conditions. *Escherichia coli* OP50 was grown on LB broth at 37°C overnight.

### *Lactobacillus delbrueckii* Subspecies Identification by ARDRA

Total bacterial DNA extraction and 16S rDNA amplification were performed as previously described (Zanni et al., 2015). ARDRA analysis was conducted by digestion of amplified 16S rDNA with EcoRI or Tru9I restriction endonucleases (Promega Italia, Milan, Italy), according to manufacturer’s instructions.

### Cellular Extraction Procedure

Cultured bacterial cells grown as described above were washed three times with cold H_2_O_dd_ and suspended in 900 μL of cold methanol (-20°C) to quench intracellular metabolism. In the meantime an aliquot of the cultures was washed and the wet weight calculated. To extract the metabolites and proteins the method reported in (Miccheli et al., 1988) was followed. The method allowed the separation of polar, organic phases and protein pellets, which were separately dried under N_2_ flux and stored at -80°C until further analysis.

### NMR Analysis

The freeze-dried polar samples were re-dissolved in 600 μL of D_2_O phosphate buffer solution (pH 7.4) containing 2 mM sodium 3-(tri-methylsilyl)propionate-2,2,3,3-d_4_ (TSP) as ^1^H NMR reference, and transferred to 5 mm NMR glass tubes for analysis. The organic phases were re-dissolved in 600 μL of CDCl_3_ containing 2 mM hexamethyl-di-siloxane (HMDSO) as a ^1^H NMR reference.

#### NMR Spectroscopy and Data Analysis

^1^H NMR spectra were acquired at 25°C using a Bruker Avance III 400 spectrometer (Bruker BioSpin GmbH, Germany) equipped with a magnet operating at 9.4 Tesla, where the ^1^H nucleus resonates at 400.13 MHz. The probe-head was a 5 mm diameter multinuclear PABBO BB-1H/D (Z108618/0044) equipped with z-gradient.

The pulse sequence adopted for spectra acquisition was a presaturation–single 90 detection pulse–acquire–delay sequence where the D1 relaxation delay was optimized to 2.5 s to allow the acquisition of 64 k data point in about 5.5 s, satisfying full relaxation conditions.

The length of the detection pulse was calibrated previously to the acquisition of each spectrum, the spectral width was set to 6009.62 Hz (15 ppm) and 64 scans were collected for each spectrum.

^1^H NMR spectra were processed using the 1D-NMR Manager ver. 12.0 software (Advanced Chemistry Development, Inc., Toronto, ON, Canada).

The assignment of the peaks to specific metabolites was achieved by standard two-dimensional (2D)^1^H-^1^H total correlation spectroscopy (TOCSY), ^1^H-^13^C heteronuclear single quantum correlation (HSQC), and heteronuclear multiple bond correlation (HMBC) and confirmed using an internal library of compounds, in comparison with literature data (Fan and Lane, 2008; Brasili et al., 2013; Gorietti et al., 2014).

The acquired NMR spectra were manually phased and baseline corrected; polar and organic spectra were referenced to the chemical shift of the TSP or HMDSO methyl resonance at δ 0.00 and 0.055 ppm, respectively. The quantification of metabolites was obtained by comparison of the integrals of specific signals to the internal standard (TSP or HMDSO) integral.

### Proteomic Analysis

Total extract of each *Lactobacillus* samples was resuspended in 200 μl of Ammonium Bicarbonate 0.1 M pH 8 and then homogenized.

After the addiction of Rapigest 0.2% v/v (Waters Corporation, Milford, MA, United States) and after incubation at 100°C for 15 min, protein content was quantified using SPN-Protein Assay (G-Biosciences, United States) and 50 μg of each sample was digested with trypsin (1:50) at 37°C o/n (Sequencing Grade Modifier Trypsin, Promega). A second aliquot of trypsin was added to samples and after 4h the digestion was stopped with TFA 0.5% (v/v). Finally, samples were incubated for 40 min at 37°C, centrifuged at 13 000 × *g* for 10 min in order to precipitate Rapigest and desalted by PepClean C-18 spin column (Pierce Biotechnology, Rockford, IL, United States).

Trypsin digested samples were analyzed by means of two-dimensional micro liquid chromatography coupled to linear ion trap mass spectrometer LTQ (Thermo Fisher Scientific, Waltham, MA, United States) as described in Comunian et al. (2011) with some modifications. Briefly, the separation of peptides was obtained through an acetonitrile gradient (eluent A, 0.1% formic acid in water; eluent B, 0.1% formic acid in acetonitrile) and the gradient profile was 5–10% eluent B in 5 min, 10–40% B in 40 min, 40–80% B in 8 min, and 80–95% in 3 min.

The mass spectrometry proteomics data have been deposited to the ProteomeXchange Consortium via the PRIDE (Vizcaíno et al., 2016) partner repository with the dataset identifier PXD006551.

### *C. elegans* Strains and Growth Conditions

The wild-type *C. elegans* strain N2 was used in all experiments and propagated on nematode growth medium (NGM) modified to be peptone-free (mNGM) (Zanni et al., 2015). Worms were fed with bacterial cultures, daily prepared as follows: aliquots of frozen bacteria stock were inoculated in Elliker broth (DIFCO) and grown at 37°C overnight under anaerobic conditions. Afterward, 25 μL of each bacterial suspension in M9 buffer, corresponding to 10 mg of bacterial cells, was spread on 3.5 cm diameter mNGM plates.

### Lifespan Assays

Synchronized N2 adults were allowed to lay embryos for 2 h directly on mNGM, covered with the indicated bacterial lawns, and then sacrificed. All lifespan assays started when the progeny became fertile (t0). Animals were transferred to new plates spread with fresh lawns and monitored daily. They were scored as dead when they no longer responded to gentle prodding with a platinum wire. Worms that crawled off the plates were not included in the analysis.

### Brood Size Measurement

Progeny production was evaluated according to (Zanni et al., 2015), with some modifications. Briefly, synchronized worms obtained as above were grown on mNGM plates seeded with bacteria and then were allowed to lay embryos at 16°C. Next, animals were transferred onto a fresh bacteria plate every day, and the number of progeny was counted with a Zeiss Axiovert 25 microscope. The procedure was repeated for 4 days until the mother worms stopped laying eggs. Each day the progeny production was recorded.

### Body Size Measurement

Individual animals were photographed after 3, 4, 5, and 6 days from egg hatching using a Leica MZ10F stereomicroscope connected to Jenoptik CCD camera. Length of worm body was determined by using the Delta Sistemi IAS software. At least 30 nematodes were imaged on at least three independent experiments.

### Estimation of Bacterial CFU within the Nematode Gut

For each experiment, 10 animals at L4 stage and at 5 days of adulthood were washed and lysed according to (Uccelletti et al., 2010). Whole worm lysates were plated onto MRS-agar plates. The number of CFU was counted after 48 h of incubation at 37°C, anaerobically.

### Statistical Analysis

Experiments were performed at least in triplicate. Data are presented as mean ± SD, and Student’s-test or one-way ANOVA analysis coupled with a Bonferroni post test (GraphPad Prism 4.0 software) was used to determine the statistical significance between experimental groups. Statistical significance was defined as ^∗^*P* < 0.05, ^∗∗^*P* < 0.01, and ^∗∗∗^*P* < 0.001.

In the case of metabolomics analysis, multivariate data analysis was carried out using Unscrambler 9.8 Software (CAMO, Oslo, Norway). Spectral data were mean-centered and autoscaled before analysis. Principal components analysis (PCA) was used to explore inherent clustering, to identify outliers and significant metabolites in the separation between sample groups.

*U* Mann–Whitney test was also applied; a *P*-value < 0.05 was considered for a statistically significant difference between sample groups.

For proteomic analysis, the experimental mass spectra produced by MudPIT analyses were correlated to tryptic peptide sequences by comparing with theoretical mass spectra, obtained by *in silico* digestion of a protein database downloaded from the NCBI website^1^ containing *Lactobacillus delbrueckii* subsp. *bulgaricus* and *Lactobacillus delbrueckii* subsp. *lactis* sequences. Data processing was performed using the 3.3.1. Bioworks version, based on SEQUEST algorithm (University of Washington, licensed to Thermo Finnigan Corp., San Josè, CA, United States), and the following parameters: Xcorr scores greater than 1.5 for singly charged peptide ions and 2.0 and 2.5 for doubly and triply charged ions, respectively, the peptide probability ≤ 0.001 and the protein consensus score value ≥ 10. These filters guaranteed that the resulting proteins have a probability value *p* ≤ 0.001. Data were treated with an in-house algorithm called MAProMa (Mauri and Deho, 2008) (Multidimensional Algorithm Protein Map), in particular a tool of MAProMa permits the comparison of the protein list obtain from the analysis of the samples. Proteins with significant differences in level, were identified by other two tools of MAProMA: DAve (Differential Average) and DCI (Differential Coefficient Index) (Mauri et al., 2005). These two algorithms are based on score values assigned by SEQUEST software to each identified protein in samples to be compared. Specifically, DAve is an index of the relative ratio between control and mutant and DCI is an index to evaluate the absolute variation of score value of each protein. Briefly, using MAProMA each identified protein in the two samples were aligned and then DAve and DCI indexes were calculated for all proteins. The threshold values imposed were very stringent: DAve > 0.4 and DAve < –0.4, DCI > 400 and DCI < –400. To increase the confidence, it is necessary that both indexes, DAve and DCI, satisfy these thresholds.

Hierarchical clustering was performed applying Ward’s method and Euclidean’s distance metric (Zhao and Karypis, 2005).

## Results

### Identification of the Foodborne *Lactobacillus delbrueckii* Strain Subspecies

**FIGURE 1:**
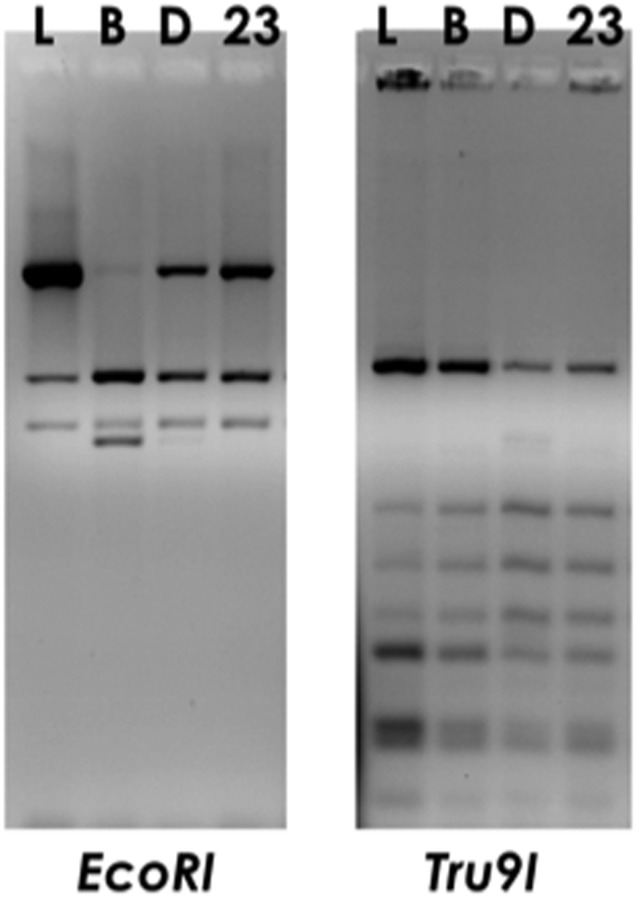
ARDRA profiles of foodborne isolate *Lactobacillus delbrueckii* 23 (23) obtained by digestion of PCR-amplified 16S rDNA with EcoRI or Tru9I restriction enzymes. Commercial isolates *L. delbrueckii* subsp. *lactis* LMG6401 (L), subsp. *bulgaricus* (B) or subsp. *delbrueckii* LMG6412T (D) were used as reference subspecies controls.

The strain *Lactobacillus delbrueckii* 23 used in this work was originally isolated from the fermented cheese MBC (Devirgiliis et al., 2008; Zanni et al., 2015), however, it had not been characterized at the subspecies level. To this aim, restriction analysis of amplified 16S ribosomal DNA (ARDRA) was performed by using endonucleases EcoRI, which allowed to differentiate subspecies *bulgaricus* from subspecies *lactis*/*delbrueckii* (Miteva et al., 2001), and Tru9I, which was used to discriminate between subspecies *delbrueckii* from subspecies *lactis*/*bulgaricus*. The results shown in **Figure 1** demonstrated that the strain *L. delbrueckii* 23 belongs to *lactis* subspecies, as revealed by the comparison of restriction patterns with reference *L. delbrueckii* subspecies.

### Proteomic Analysis of *L. delbrueckii* Subspecies

**FIGURE 2:**
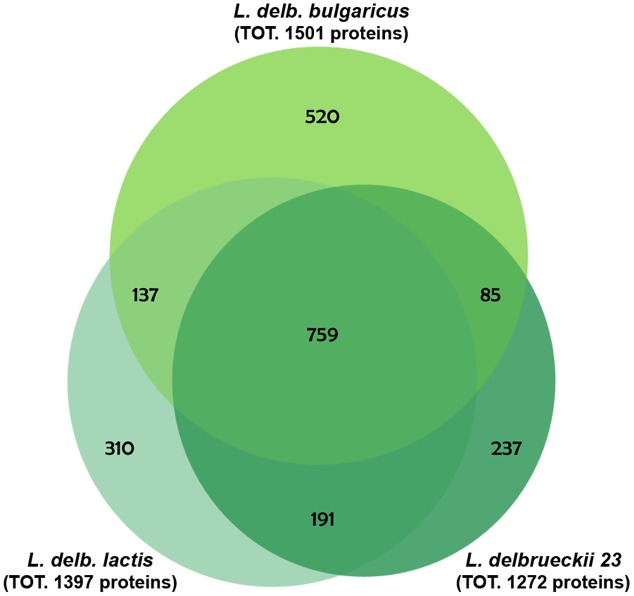
Venn diagram of protein distribution across the analyzed strains. Proteins are identified from total cell extracts derived from lactobacilli grown overnight in Elliker broth.

To highlight differences among the three strains belonging to *L. delbrueckii* subspecies relevant in dairy technologies, a proteomic analysis of *L. delbrueckii* subsp. *bulgaricus, lactis* and *L. delbrueckii* 23 was performed, using protein pellets from bacterial cells grown on Elliker’s broth, as specified in “Materials and Methods.” A total of 2239 proteins were identified in all samples (Supplementary Table S1), whose distribution across strains was analyzed by Venn diagram (**Figure 2**). *L. delbrueckii* subsp. *lactis* and *L. delbrueckii* 23 shared 191 proteins, while 137 and 85 proteins were shared by these strains with *L. delbrueckii bulgaricus*, respectively. A total of 759 proteins resulted common to all the strains, whereas 520, 310, and 237 proteins were specific to *L. delbrueckii* subsp. *bulgaricus, lactis* and *L. delbrueckii* 23, respectively (**Figure 2**).

**FIGURE 3:**
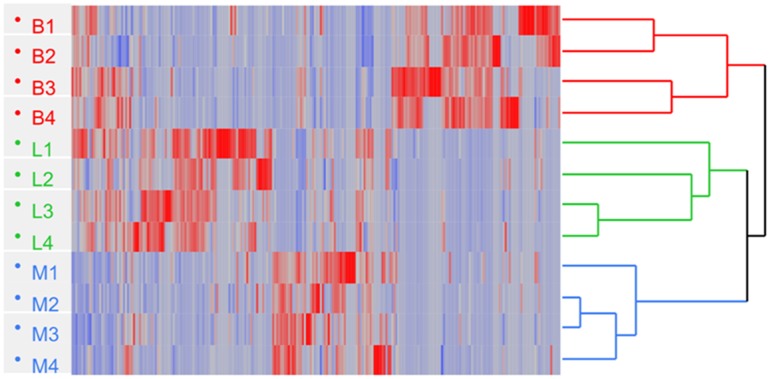
Hierarchical clustering of *L. delbrueckii* subsp. *bulgaricus* (B; *n* = 4), *lactis* (L; *n* = 4), and *L. delbrueckii* 23 (M; *n* = 4) obtained by processing the SpC (Spectral Count) of proteins; Euclidean’s distance and Ward method were applied. Heat map shows the SpC and indicates down (blue) and up-regulated (red) proteins, respectively.

To further analyze the abundance profiles of the significantly changed proteins that were commonly identified in the three strains, hierarchical clustering analysis was performed. The two strains of *L. delbrueckii* subsp. *lactis* resulted closer with respect to *L. delbrueckii* subsp. *bulgaricus*, although differences among them were detected (**Figure 3**).

The majority of differentially expressed proteins were related to bacterial metabolism. In particular, proteins exclusively expressed by *L. delbrueckii* subsp. *bulgaricus* appeared to be related to aminoacid metabolism, those exclusively expressed by *L. delbrueckii* subsp. *lactis* were related to glutathione and maltose metabolism, while sugar metabolism and fatty acid biosynthesis were specific to the strain *L. delbrueckii* 23 (Supplementary Table S2). In particular, enzymes involved in galactose metabolism through the tagatose pathway were specifically identified in such strain (Supplementary Figure S1). It is worth noting that dihydropteroate synthase as well as GTP cyclohydrolase, key enzymes of folate biosynthetic pathway, were upregulated in *L. delbrueckii* subsp. *bulgaricus* (Supplementary Figure S2).

### NMR Analysis of *L. delbrueckii* Subspecies

In order to further explore the differences among the three strains in terms of metabolites, a ^1^H-NMR spectroscopy analysis was performed. To this aim, an NMR-based metabolic profiling from aqueous as well as organic cellular extract phases was generated from the same bacterial cell samples used for proteomic experiments.

In Supplementary Table S3, the resonance assignments of the metabolites measured on polar and organic extracts are depicted. A representative ^1^H NMR spectrum of cell polar extracts was reported in Supplementary Figure S3. The analysis of intracellular metabolites reported in **Table 1** revealed the presence of higher amounts of several amino acids in *L. delbrueckii* subsp. *bulgaricus*.

**Table 1:** Amount of intracellular metabolites expressed as μmol/g calculated as the average of three samples +/- standard deviation.

	*L. delbrueckii bulgaricus*	*L. delbrueckiilactis*	*L. delbrueckii* 23	Significativity
**Aminoacids**				
Valine	0.28 ± 0.07	0.25 ± 0.02	0.47 ± 0.05	C
Isoleucine	0.22 ± 0.05	0.16 ± 0.03	0.30 ± 0.04	C
Leucine	0.34 ± 0.06	0.35 ± 0.04	0.75 ± 0.08	BC
Threonine	0.46 ± 0.00	1.30 ± 0.28	0.33 ± 0.05	AC
Alanine	0.59 ± 0.00	0.40 ± 0.06	0.64 ± 0.16	
Glutamate	9.17 ± 0.77	1.70 ± 0.28	2.26 ± 0.56	AB
Glutamine	2.42 ± 0.05	0.02 ± 0.00	1.34 ± 0.51	AC
Glutathione	0.00 ± 0.00	0.16 ± 0.02	0.54 ± 0.10	ABC
Aspartate	5.18 ± 0.16	1.07 ± 0.19	1.35 ± 0.64	AB
Asparagine	2.47 ± 0.41	0.34 ± 0.08	0.94 ± 0.39	ABC
Lysine	2.88 ± 0.03	1.27 ± 0.33	5.23 ± 0.40	ABC
Arginine	0.11 ± 0.07	7.25 ± 0.93	0.39 ± 0.09	ABC
Serine	6.42 ± 0.08	0.00 ± 0.00	0.00 ± 0.00	AB
Glycine	0.61 ± 0.00	0.21 ± 0.02	0.35 ± 0.05	AB
Tyrosine	0.17 ± 0.05	0.04 ± 0.01	1.79 ± 1.39	BC
Histidine	0.19 ± 0.11	0.29 ± 0.16	0.14 ± 0.05	
Phenylalanine	0.04 ± 0.02	0.08 ± 0.02	0.16 ± 0.02	
Tryptophan	0.08 ± 0.05	0.05 ± 0.02	0.08 ± 0.03	
**Organic acids**				
Lactate	0.74 ± 0.07	0.25 ± 0.02	15.83 ± 1.82	ABC
Acetate	0.53 ± 0.03	0.97 ± 0.19	1.32 ± 0.08	AB
Formate	0.19 ± 0.00	0.36 ± 0.19	0.29 ± 0.04	
**Carbohydrates**				
Glucose	1.53 ± 0.81	0.00 ± 0.00	0.00 ± 0.00	AB
**Fatty Acids**				
Saturated fatty acid	1.32 ± 0.14	2.77 ± 0.18	2.51 ± 0.21	AB
Monoinsaturated fatty acid	7.82 ± 1.08	5.97 ± 1.17	2.18 ± 0.17	BC
Monoacyl glycerol	2.26 ± 0.05	2.73 ± 0.11	2.07 ± 0.12	
**Miscellaneous**				
Dilactate	0.26 ± 0.00	0.07 ± 0.01	0.06 ± 0.02	AB
Choline	0.00 ± 0.00	3.72 ± 0.53	0.09 ± 0.02	AC
Uridine Phosphate	1.42 ± 0.16	2.89 ± 0.29	0.59 ± 0.11	ABC
Cytosine Phosphate	0.73 ± 0.05	0.64 ± 0.09	0.34 ± 0.05	
Guanidine Phosphate	0.56 ± 0.08	0.69 ± 0.13	0.90 ± 0.10	
Adenine Phosphate	0.31 ± 0.05	0.55 ± 0.07	0.12 ± 0.03	C
NAD	0.50 ± 0.05	0.74 ± 0.08	0.90 ± 0.12	


*Univariate statistical analysis was performed employing the non-parametrical *U* Mann–Whitney test. The letters A, B, and C indicate significant statistical differences (*p* < 0.05) with the code A indicating a discrimination between *L. delbrueckii bulgaricus* and *L. delbrueckii lactis*, B between *L. delbrueckii bulgaricus* and *L. delbrueckii* 23, and C between *L. delbrueckii lactis* and *L. delbrueckii* 23.*

Concerning fat content, saturated fatty acids (SFA) resulted lower in *L. delbrueckii* subsp. *bulgaricus*, while the monounsaturated fatty acids were higher. Intracellular glucose was detected only in this subspecies, whereas glutathione was present only in *L. delbrueckii* subsp. *lactis*, although at different amounts in the two strains. Finally, dilactic acid showed a higher intracellular concentration in *L. delbrueckii* subsp. *bulgaricus* (**Table 1**).

**FIGURE 4:**
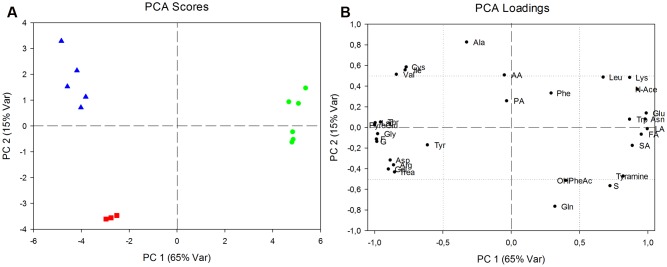
Analysis of production and consumption of metabolites, expressed by net balances. **(A)** Principal components analysis (PCA) score plot relative to *L. delbrueckii* 23 (green circles), *L. delbrueckii bulgaricus* (red squares) and *L. delbrueckii lactis* (blue triangles). Eighty percent of total variability represented in two components (PC1: 65%; PC2: 15%). **(B)** PCA loadings plot indicating statistically significant differences of excreted or consumed metabolites between the two *L. delbrueckii* 23 and *L. delbrueckii lactis* with respect to *L. delbrueckii bulgaricus* along PC1, or between both *L. delbrueckii lactis* subspecies and *L. delbrueckii bulgaricus* along PC2.

The analysis of synthesis and consumption of metabolites provided more information on specific metabolism of the single subspecies than intracellular content. Principal component analysis was performed to explore the data field (**Figure 4**).

Component scores plot showed along PC1 a net separation between *L. delbrueckii* 23 and *L. delbrueckii* subsp. *lactis* together with *L. delbrueckii* subsp. *bulgaricus*; whereas a separation between *L. delbrueckii* subsp. *lactis* strains and *L. delbrueckii* subsp. *bulgaricus* was showed along PC2.

The data, reported in **Table 2**, are representative of net balances, with positive and negative values being considered as an estimate of net fluxes of production and utilization of metabolites, respectively. Both aminoacid and sugar metabolism revealed differences among the three isolates, and in particular between the two strains of *L. delbrueckii* subsp. *lactis*. Interestingly, all the three strains produced aminoacids such as leucine, alanine, aspartate, arginine and aromatic aminoacids and consumed glutamate. Conversely, threonine, glutamine, pyroglutamate, cysteine, and asparagine displayed opposite delta values between the two *L. delbrueckii lactis* strains, and in the case of valine, glutamic acid and glycine the amounts of produced/consumed metabolites, although displaying the same trend, were significantly different between the two strains. However, the sugar metabolism resulted to be particularly strain-specific. *L. delbrueckii* 23 showed a strong ability to metabolize lactose, galactose, fructose, and trealose with a parallel production of high amounts of lactic acid. It is worth noting that *L. delbrueckii* subsp. *lactis* and *L. delbrueckii* subsp. *bulgaricus* produced glucose and galactose, although this latter component was detected at lower levels in the case of *lactis* strain. These sugars derived from the incomplete catabolism of lactose by these two lactobacilli being not able to further metabolize galactose. As a consequence, the sugars were secreted out of the cell, probably through lactose-galactose antiporter system. Sucrose was consumed only by *L. delbrueckii* subsp. *lactis*, while glucose was consumed only by *L. delbrueckii* 23.

**Table 2:** Medium balances expressed as mM and calculated as the average of three samples +/- standard deviation.

	*L. delbrueckii bulgaricus*	*L. delbrueckii lactis*	*L. delbrueckii 23*	Significativity
**Aminoacids**			
Valine	0.55 ± 0.02	1.31 ± 0.10	0.26 ± 0.06	A^∗^BC^∗^
Isoleucine	-1.00 ± 0.01	-0.64 ± 0.03	-1.11 ± 0.06	C
Leucine	1.46 ± 0.04	1.97 ± 0.18	3.07 ± 0.40	B
Threonine	1.18 ± 0.16	1.61 ± 0.13	-0.30 ± 0.11	B^∗^C^∗^
Alanine	0.44 ± 0.01	0.99 ± 0.02	0.65 ± 0.11	A
Lysine	-3.23 ± 0.03	-1.97 ± 0.12	0.16 ± 0.19	A^∗^B^∗^C^∗^
Arginine	10.93 ± 0.11	9.63 ± 0.04	7.46 ± 0.21	A^∗^B^∗^C^∗^
Pyroglutamate	6.89 ± 0.01	8.53 ± 0.09	-0.02 ± 0.21	B^∗^C^∗^
Glutamate	-8.15 ± 0.01	-8.14 ± 0.01	-1.67 ± 0.09	B^∗^C^∗^
Glutamine	1.97 ± 0.04	-0.43 ± 0.13	0.83 ± 0.19	A^∗^BC
Aspartate	12.92 ± 0.34	11.78 ± 0.37	8.56 ± 0.21	AB^∗^C^∗^
Asparagine	-1.52 ± 0.09	-1.67 ± 0.12	0.62 ± 0.11	B^∗^C^∗^
Cysteine	-1.45 ± 0.34	1.98 ± 0.27	-1.84 ± 0.17	A^∗^C^∗^
Glycine	-1.46 ± 0.16	-1.41 ± 0.53	-8.57 ± 0.19	B^∗^C^∗^
Tyrosine	1.09 ± 0.05	1.07 ± 0.09	0.86 ± 0.03	
Phenylalanine	0.92 ± 0.07	1.09 ± 0.14	1.18 ± 0.13	
Tryptophan	0.60 ± 0.06	0.64 ± 0.19	1.46 ± 0.06	B^∗^C^∗^
**Organic Acids**			
Lactate	50.62 ± 0.34	37.26 ± 2.48	106.12 ± 1.55	A^∗^B^∗^C^∗^
Acetate	4.54 ± 0.06	5.21 ± 0.73	4.93 ± 0.32	
*N*-Acetyl	1.47 ± 0.03	1.73 ± 0.02	2.52 ± 0.06	B
Pyruvate	0.84 ± 0.01	1.09 ± 0.16	0.98 ± 0.02	
Succinate	0.55 ± 0.01	0.39 ± 0.09	0.85 ± 0.02	BC^∗^
Formate	0.51 ± 0.02	0.05 ± 0.21	1.75 ± 0.05	C
**Carbohydrates**			
Sucrose	0.63 ± 0.19	-2.62 ± 0.18	0.63 ± 0.22	A^∗^C^∗^
Fructose	-3.23 ± 0.23	-3.11 ± 0.14	-8.67 ± 0.01	A
Lactose	-4.33 ± 0.13	-0.71 ± 0.23	-16.11 ± 0.02	C
Galactose	3.91 ± 0.18	0.86 ± 0.42	-5.65 ± 0.01	B
Trealose	-0.26 ± 0.09	-0.62 ± 0.11	-1.37 ± 0.01	B
Glucose	5.79 ± 0.29	5.77 ± 0.18	-0.89 ± 0.01	BC^∗^
**Miscellaneous**			
Tyramine	0.37 ± 0.01	0.16 ± 0.04	0.44 ± 0.02	A^∗^C^∗^
4-Hydroxyphenylacetate	0.19 ± 0.02	0.02 ± 0.10	0.18 ± 0.01	AC


*A positive value corresponds to the production of a metabolite compared to the starting medium, while a negative one indicates consumption. The letters A, B, and C indicate significant statistical (*p* < 0.05) differences according to ANOVA on ranks analysis with the code A discriminating between *L. delbrueckii bulgaricus* and *L. delbrueckii lactis*, B between *L. delbrueckii bulgaricus* and *L. delbrueckii* 23, and C between *L. delbrueckii lactis* and *L. delbrueckii* 23. Asterisks indicate the discriminations with a ox*p*-value < 0.01.*

### Evaluation of Potential Probiotic Effects of *L. delbrueckii* Subspecies in *C. elegans*

**FIGURE 5:**
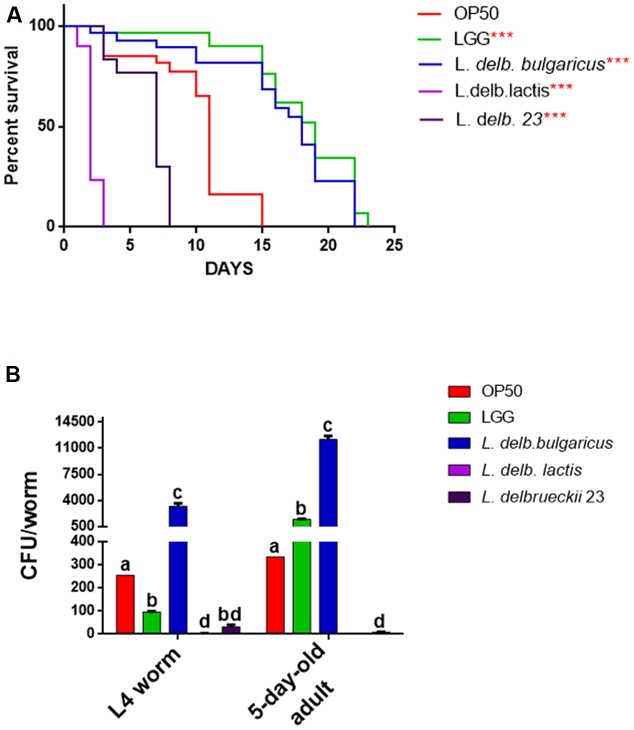
Effect of *L. delbrueckii* subspecies on nematode lifespan and gut colonization capacity. **(A)** Kaplan–Mèier survival plot of N2 worms fed the three *L. delbrueckii* strains or controls (LGG and *Escherichia coli* OP50); *n* = 60 for each data point of single experiments. Asterisks indicate the *P*-values (log-rank test) with respect to the control *E. coli* OP50. Different letters indicate statistically significant differences (*P* < 0.05). **(B)** Bacterial colony forming units (CFU) recovered from nematodes, obtained by plating whole lysates of L4 and 5-days adults fed the three *L. delbrueckii* strains or controls (LGG and *E. coli* OP50). Bars represent the mean of three independent experiments.

We next aimed at understanding possible probiotic effects exerted by these isolates by using the model organism *C. elegans*. To this purpose, phenotypical analysis was performed on animals grown on the *L. delbrueckii* strains. Notably, the dietary administration of *L. delbrueckii* subsp. *bulgaricus* led to an increased worm longevity with respect to both the commercial and the foodborne isolate *L. delbrueckii* subsp. *lactis* (**Figure 5A**).

Indeed, a median survival of 18 days was found in animals fed with *L. delbrueckii* subsp. *bulgaricus*, while a median survival of 11 days was observed in worms fed with the standard food source OP50. Moreover, the use of a claimed probiotic strain, namely LGG, as a food source, resulted in an almost identical effect on worm longevity. On the other hand, median survival values obtained for nematodes fed with *L. delbrueckii* subsp. *lactis* and *L. delbrueckii* 23 strains were recorded at 2 and 7 days, respectively. In addition, bacterial colonization capacity, a feature strongly related to probiotic activity, was assessed at L4 and 5-days adulthood stages in animals fed with the different bacteria (**Figure 5B**). In L4 worms, *L. delbrueckii* subsp. *bulgaricus* showed the maximum colonization capacity, expressed as CFU/worm; about 30-fold higher with respect of the probiotic control LGG. By contrast, the two strains of *L. delbrueckii* subsp. *lactis* exhibited almost undetectable CFU/worm values, which resulted even lower with respect to OP50 control (**Figure 5B**). In 5-days adult nematodes the same trend was observed, with *L. delbrueckii* subsp. *bulgaricus* and LGG showing the maximum colonization capacity, although with a less marked relative difference. Moreover, *L. delbrueckii* 23 CFU/worm decreased by four times with respect to the earlier animal stage (**Figure 5B**).

**FIGURE 6:**
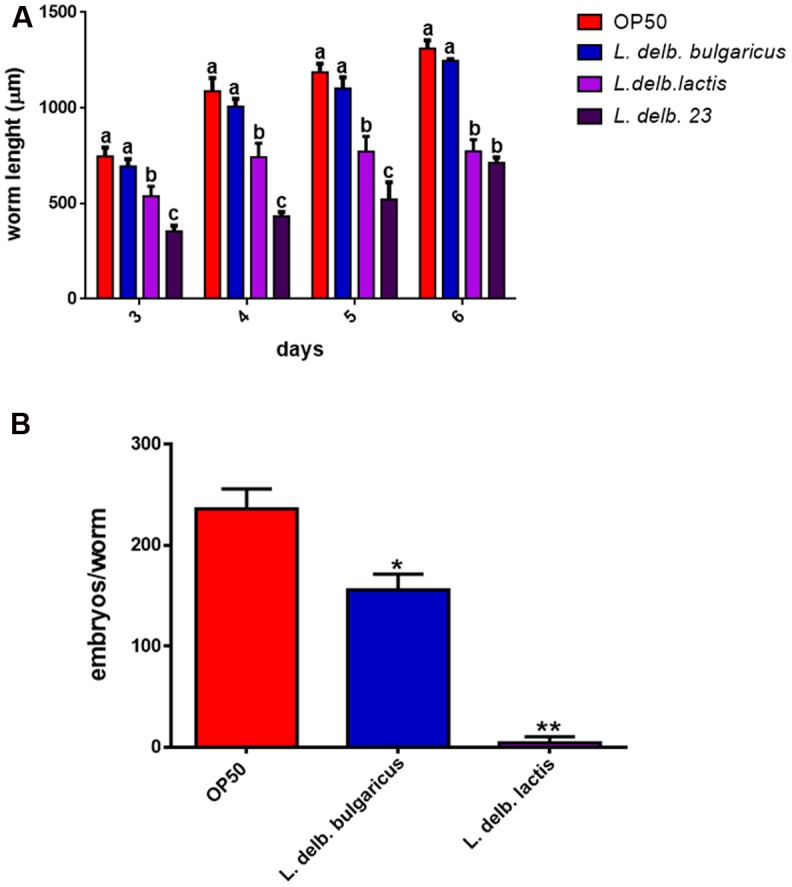
Analysis of body size and fertility rate in worms fed *L. delbrueckii* subspecies. **(A)** Effect on *Caenorhabditis elegans* body size. Worms were grown in the presence of the three *L. delbrueckii* strains or OP50, their length was measured from head to tail at the indicated time points. Different letters indicate statistically significant differences (*P* < 0.05). **(B)** Average embryos production per worm of *L. delbrueckii* strains-fed animals. (^∗^*P* < 0.05; ^∗∗^*P* < 0.01).

In order to evaluate other physiological effects exerted by *L. delbrueckii* strains, analysis of larval development was conducted. *L. delbrueckii* subsp. *bulgaricus* did not influence the worm length, while reduction in measures was observed in animals fed with both *L. delbrueckii lactis* strains (**Figure 6A**).

Finally, nematode fertility was also evaluated, through brood size analysis, expressed as number of embryos/worm. A slight reduction of progeny production was observed in worms fed with *L. delbrueckii* subsp. *bulgaricus* with respect to those fed with OP50, whereas a very low number of embryos laid by animals grown on *L. delbrueckii* subsp. *lactis* was recorded (**Figure 6B**). *L. delbrueckii* 23 diet induced a sterile phenotype in the nematodes (data not shown).

## Discussion

In the present work a multidisciplinary approach was applied to characterize three strains belonging to *L. delbrueckii* subspecies *bulgaricus* and *lactis*, by combining metabolomic and proteomic analysis as well as *in vivo* screening in the model organism *C. elegans*, with the final aim to evaluate their possible probiotic effects.

In particular, one of the strains, namely *L. delbrueckii* 23, was newly isolated from the fermenting microbiota of a traditional fermented fresh cheese (Zanni et al., 2015), while the other two strains, belonging to the subspecies *bulgaricus* and *lactis*, were acquired from bacterial culture collections ATCC and LMG, respectively. ARDRA analysis performed on *L. delbrueckii* 23 revealed that this strain belonged to subspecies *lactis*.

Both proteomic and metabolomic approaches showed differences among the three isolates. In particular, hierarchical cluster analysis of proteomic data highlighted that the two strains of *L. delbrueckii* subsp. *lactis* resulted closer with respect to *L. delbrueckii* subsp. *bulgaricus*, even if differences among them were detected. NMR spectroscopy-metabolomic profiles obtained for the *L. delbrueckii* subspecies highlighted different metabolome profiles among the three isolates. We have considered both the intracellular metabolites as well as the specific metabolism of the single subspecies, this latter through the analysis of synthesis and consumption of metabolites, expressed by net balances of extracellular metabolites. In particular, the obtained results showed that metabolism of *L. delbrueckii* subsp. *bulgaricus* and *lactis* are mainly sustained from a protein-based source, on the contrary, the *L. delbrueckii* 23 strain appeared to be mainly dependent on carbohydrate metabolism. Analysis of sugar metabolism showed differences between the two strains of *L. delbrueckii* subsp. *lactis*, in particular concerning galactose metabolism, in agreement with the proteomic experiments. Intriguingly, the newly isolated strain *L. delbrueckii* 23 resulted capable of metabolizing galactose through the tagatose pathway, a trait that could reflect its origin from MBC. In fact, most strains of *L. delbrueckii* subsp. *bulgaricus*, used as starter cultures for Mozzarella cheese, ferment only the glucose portion of lactose and release the galactose portion into the medium or curd, whereas *L. delbrueckii* subsp. *lactis* strains can metabolize, in addition, galactose (Suzzi et al., 2000; Germond et al., 2003). This avoids accumulation of the galactose in dairy products, which may lead to several undesirable effects, such as browning of Mozzarella cheese (Vaillancourt et al., 2004).

Furthermore, we took advantage of the simple model organism *C. elegans* to perform *in vivo* analysis in order to evaluate the potential probiotic effects exerted by the three *L. delbrueckii* strains. Our findings demonstrated that feeding worms with different *L. delbrueckii* subspecies results in opposite effects on host metabolism. In particular, *L. delbrueckii* subsp. *bulgaricus* exerted probiotic features in terms of life-span extension and gut colonization capacity, whereas the two strains belonging to *L. delbrueckii* subsp. *lactis* impacted more severely on lifespan and larval development. One of the key aspects in determining these different behaviors could be represented by the gut colonization capacity, since *L. delbrueckii* subsp. *bulgaricus* resulted capable of colonizing the worm’s gut more efficiently with respect to the subsp. *lactis*. According to our hypothesis, Park et al. (2014) observed that newly isolated strains of *L. plantarum*, which were more efficient in colonizing the nematode gut with respect to LGG, were able to extend the worm’s lifespan. However, this aspect is still controversial, since other authors reported a negative correlation between colonization capacity and lifespan extension (Brokate-Llanos et al., 2014; Park et al., 2015), or suggested that gut colonization does not directly influence the host defense response (Yun et al., 2014). It must be pointed out that in the case of *E. coli* as food source, the reduction of colonization capacity could lower bacterial pathogenicity resulting in an increased worm’s longevity (Brokate-Llanos et al., 2014). Moreover, it has been observed that the effects of bacterial feeding on worm longevity are often strain-specific (Park et al., 2015).

It has been demonstrated that bacterial metabolism plays a key role in affecting nematode longevity (Nguyen and Clarke, 2012; Brokate-Llanos et al., 2014; Yu et al., 2015). A trait characterizing *L. delbrueckii* subsp. *bulgaricus* strain was represented by its ability to synthesize folate, as indicated by proteomic analysis. Bacterial folate metabolism has received great consideration in terms of influencing *C. elegans* life span, in particular correlating bacterial folate synthesis inhibition with reduction of worm longevity (Virk et al., 2012). A recent paper clarified that bacterial folate status acts on *E. coli* physiology in a way that influences worm aging (Virk et al., 2016). To the best of our knowledge, however, this kind of studies have never been conducted in alternative worm’s food sources, such as LAB.

Analysis of amino acid metabolism revealed that serine, glycine, aspartic acid, asparagine, and glutamic acid accumulated in *L. delbrueckii* subsp. *bulgaricus* cells, whereas their levels were lower in the two *lactis* strains. Aminoacid metabolism has been shown to influence lifespan, in particular tryptophan and proline, among others, were associated with longevity, while aspartic acid and phenylalanine seem to have anti-longevity effects (Edwards et al., 2015). It could be likely that the differentially accumulated aminoacids in the two subspecies can contribute to the effects exerted on host lifespan. Concerning fat metabolism, both strains of *L. delbrueckii* subsp. *lactis* exhibited higher intracellular levels of SFA than *L. delbrueckii* subsp. *bulgaricus*. According to our results, Kudo et al. (2012) found that the content of several SFA, including palmitic (C:16) and stearic (C:18) acids, was increased in *L. delbrueckii* subsp. *lactis* with respect to *L. delbrueckii* subsp. *bulgaricus*. Moreover, it has been reported that *C. elegans* lifespan was slightly reduced by supplementation of palmitic acid in the diet (Shmookler Reis et al., 2011). We can thus not exclude that the increased levels of SFA may in part contribute to the effects exerted by *L. delbrueckii* subsp. *lactis* on worm longevity.

Sugar metabolism can also be correlated to lifespan, since evidences suggest that the presence of several carbohydrates, such as glucose, galactose or lactose, derived from bacterial metabolism, increase the nematode longevity (Brokate-Llanos et al., 2014). The ability of *L. delbrueckii* subsp. *bulgaricus* to secrete galactose could be related to its beneficial effects observed in *C. elegans*.

## Conclusion

In the present work three strains belonging to *L. delbrueckii* subspecies *bulgaricus* and *lactis* were characterized applying a phenomic approach based on proteomic and metabolomic analysis as well as *in vivo* evaluation of their potential probiotic effects. Metabolic profiling highlighted the different origin of the three strains. Moreover, the *L. delbrueckii* subspecies *bulgaricus* strain demonstrated potential probiotic features. Overall, our results demonstrate that combination of *omic* platforms coupled with *in vivo* screening in the model organism *C. elegans* represents a powerful tool to characterize industrially relevant bacterial isolates.

## Author Contributions

Conceived and designed the experiments: DU, CD, and AM. Wrote the paper: CD, DU, and EZ. Critical revision of manuscript: GP and CP. Did animal experiments/treatments: EZ and ES. Performed metabolomics experiments: FS. Performed proteomics experiments: SM. Analyzed and supervised proteomics data: PM.

## Conflict of Interest Statement

The authors declare that the research was conducted in the absence of any commercial or financial relationships that could be construed as a potential conflict of interest.

## Footnotes

1 http://www.ncbi.nlm.nih.gov/Ftp/index.html

## References

[B1] Brasili E. Mengheri E. Tomassini A. Capuani G. Roselli M. Finamore A. (2013). *Lactobacillus acidophilus* La5 and *Bifidobacterium lactis* Bb12 induce different age-related metabolic profiles revealed by 1H-NMR spectroscopy in urine and feces of mice. *J. Nutr.* 143 1549–1557. 10.3945/jn.113.177105 23946343

[B2] Brokate-Llanos A. M. Garzon A. Munoz M. J. (2014). *Escherichia coli* carbon source metabolism affects longevity of its predator *Caenorhabditis elegans*. *Mech. Ageing Dev.* 14 22–25. 10.1016/j.mad.2014.09.001 25263107

[B3] Calvani R. Brasili E. Pratico G. Sciubba F. Roselli M. Finamore A. (2014). Application of NMR-based metabolomics to the study of gut microbiota in obesity. *J. Clin. Gastroenterol.* 48(Suppl. 1), S5–S7. 10.1097/MCG.0000000000000236 25291128

[B4] Clark L. C. Hodgkin J. (2014). Commensals, probiotics and pathogens in the *Caenorhabditis elegans* model. *Cell Microbiol.* 16 27–38. 10.1111/cmi.12234 24168639

[B5] Comunian C. Rusconi F. De Palma A. Brunetti P. Catalucci D. Mauri P. L. (2011). A comparative MudPIT analysis identifies different expression profiles in heart compartments. *Proteomics* 11 2320–2328. 10.1002/pmic.201000479 21598388

[B6] Dellaglio F. Felis G. E. Castioni A. Torriani S. Germond J. E. (2005). *Lactobacillus delbrueckii* subsp. indicus subsp. nov., isolated from Indian dairy products. *Int. J. Syst. Evol. Microbiol.* 55 401–404. 10.1099/ijs.0.63067-0 15653908

[B7] Devirgiliis C. Barile S. Perozzi G. (2011). Antibiotic resistance determinants in the interplay between food and gut microbiota. *Genes Nutr.* 6 275–284. 10.1007/s12263-011-0226-x 21526400 PMC3145056

[B8] Devirgiliis C. Caravelli A. Coppola D. Barile S. Perozzi G. (2008). Antibiotic resistance and microbial composition along the manufacturing process of Mozzarella di Bufala Campana. *Int. J. Food Microbiol.* 128 378–384. 10.1016/j.ijfoodmicro.2008.09.021 18990462

[B9] Edwards C. Canfield J. Copes N. Brito A. Rehan M. Lipps D. (2015). Mechanisms of amino acid-mediated lifespan extension in *Caenorhabditis elegans*. *BMC Genet.* 16:8 10.1186/s12863-015-0167-2 PMC4328591 25643626

[B10] El Kafsi H. Binesse J. Loux V. Buratti J. Boudebbouze S. Dervyn R. (2014). *Lactobacillus delbrueckii* ssp. lactis and ssp. bulgaricus: a chronicle of evolution in action. *BMC Genomics* 15:407 10.1186/1471-2164-15-407 PMC4082628 24884896

[B11] Fan W. M. T. Lane A. N. (2008). Lane Structure-based profiling of metabolites and isotopomers by NMR. *Prog. Nucl. Magn. Reson. Spectrosc.* 52 69–117. 10.1007/s10858-011-9484-6

[B12] Germond J. E. Lapierre L. Delley M. Mollet B. Felis G. E. Dellaglio F. (2003). Evolution of the bacterial species *Lactobacillus delbrueckii*: a partial genomic study with reflections on prokaryotic species concept. *Mol. Biol. Evol.* 20 93–104. 10.1093/molbev/msg012 12519911

[B13] Gorietti D. Zanni E. Palleschi C. Delfini M. Uccelletti D. Saliola M. (2014). Depletion of casein kinase I leads to a NAD(P)(+)/NAD(P)H balance-dependent metabolic adaptation as determined by NMR spectroscopy-metabolomic profile in Kluyveromyces lactis. *Biochim. Biophys. Acta* 1840 556–564. 10.1016/j.bbagen.2013.10.020 24144565

[B14] Kudo Y. Oki K. Watanabe K. (2012). *Lactobacillus delbrueckii* subsp. sunkii subsp. nov., isolated from sunki, a traditional Japanese pickle. *Int. J. Syst. Evol. Microbiol.* 62 2643–2649. 10.1099/ijs.0.037051-0 22199209

[B15] Lee J. Kwon G. Lim Y. H. (2015). Elucidating the mechanism of weissella-dependent lifespan extension in *Caenorhabditis elegans*. *Sci. Rep.* 5:17128 10.1038/srep17128 PMC4658530 26601690

[B16] Liu S. Q. (2003). Practical implications of lactate and pyruvate metabolism by lactic acid bacteria in food and beverage fermentations. *Int. J. Food Microbiol.* 83 115–131. 10.1016/S0168-1605(02)00366-5 12706034

[B17] Mauri P. Deho G. (2008). A proteomic approach to the analysis of RNA degradosome composition in *Escherichia coli*. *Methods Enzymol.* 447 99–117. 10.1016/S0076-6879(08)02206-4 19161840

[B18] Mauri P. Scarpa A. Nascimbeni A. C. Benazzi L. Parmagnani E. Mafficini A. (2005). Identification of proteins released by pancreatic cancer cells by multidimensional protein identification technology: a strategy for identification of novel cancer markers. *FASEB J.* 19 1125–1127. 10.1096/fj.04-3000fje 15985535

[B19] Miccheli A. Aureli T. Delfini M. Di Cocco M. E. Viola P. Gobetto R. (1988). Study on influence of inactivation enzyme techniques and extraction procedures on cerebral phosphorylated metabolite levels by 31P NMR spectroscopy. *Cell Mol. Biol.* 34 591–603. 3219695

[B20] Miteva V. Boudakov I. Ivanova-Stoyancheva G. Marinova B. Mitev V. Mengaud J. (2001). Differentiation of *Lactobacillus delbrueckii* subspecies by ribotyping and amplified ribosomal DNA restriction analysis (ARDRA). *J. Appl. Microbiol.* 90 909–918. 10.1046/j.1365-2672.2001.01320.x 11412321

[B21] Morea M. Baruzzi F. Cocconcelli P. S. (1999). Molecular and physiological characterization of dominant bacterial populations in traditional mozzarella cheese processing. *J. Appl. Microbiol.* 87 574–582. 10.1046/j.1365-2672.1999.00855.x 10583686

[B22] Nakagawa H. Shiozaki T. Kobatake E. Hosoya T. Moriya T. Sakai F. (2016). Effects and mechanisms of prolongevity induced by *Lactobacillus gasseri* SBT2055 in *Caenorhabditis elegans*. *Aging Cell* 15 227–236. 10.1111/acel.12431 26710940 PMC4783334

[B23] Nakayama Y. Moriya T. Sakai F. Ikeda N. Shiozaki T. Hosoya T. (2014). Oral administration of *Lactobacillus gasseri* SBT2055 is effective for preventing influenza in mice. *Sci. Rep.* 4:4638 10.1038/srep04638 PMC3982165 24717726

[B24] Nguyen T. P. Clarke C. F. (2012). Folate status of gut microbiome affects *Caenorhabditis elegans* lifespan. *BMC Biol.* 10:66 10.1186/1741-7007-10-66 PMC3409036 22849295

[B25] Park M. R. Oh S. Son S. J. Park D. J. Kim S. H. Jeong D. Y. (2015). *Bacillus licheniformis* isolated from traditional korean food resources enhances the longevity of *Caenorhabditis elegans* through serotonin signaling. *J. Agric. Food Chem.* 63 10227–10233. 10.1021/acs.jafc.5b03730 26541069

[B26] Park M. R. Yun H. S. Son S. J. Oh S. Kim Y. (2014). Short communication: development of a direct in vivo screening model to identify potential probiotic bacteria using *Caenorhabditis elegans*. *J. Dairy Sci.* 97 6828–6834. 10.3168/jds.2014-8561 25200770

[B27] Rebollar E. A. Antwis R. E. Becker M. H. Belden L. K. Bletz M. C. Brucker R. M. (2016). Using “Omics” and integrated multi-omics approaches to guide probiotic selection to mitigate chytridiomycosis and other emerging infectious diseases. *Front. Microbiol.* 7:68 10.3389/fmicb.2016.00068 PMC4735675 26870025

[B28] Ruiz L. Hidalgo C. Blanco-Miguez A. Lourenco A. Sanchez B. Margolles A. (2016). Tackling probiotic and gut microbiota functionality through proteomics. *J. Proteomics* 147 28–39. 10.1016/j.jprot.2016.03.023 27003613

[B29] Shmookler Reis R. J. Xu L. Lee H. Chae M. Thaden J. J. Bharill P. (2011). Modulation of lipid biosynthesis contributes to stress resistance and longevity of *C. elegans* mutants. *Aging (Albany NY)* 3 125–147. 21386131 10.18632/aging.100275 PMC3082008

[B30] So S. Tokumaru T. Miyahara K. Ohshima Y. (2011). Control of lifespan by food bacteria, nutrient limitation and pathogenicity of food in *C. elegans*. *Mech. Ageing Dev.* 132 210–212. 10.1016/j.mad.2011.02.005 21354440

[B31] Suzzi G. Lombardi A. Lanorte M. T. Caruso M. Andrighetto C. Gardini F. (2000). Phenotypic and genotypic diversity of yeasts isolated from water-buffalo Mozzarella cheese. *J. Appl. Microbiol.* 88 117–123. 10.1046/j.1365-2672.2000.00926.x 10735250

[B32] Tamang J. P. Watanabe K. Holzapfel W. H. (2016). Review: diversity of microorganisms in global fermented foods and beverages. *Front. Microbiol.* 7:377 10.3389/fmicb.2016.00377 PMC4805592 27047484

[B33] Uccelletti D. Zanni E. Marcellini L. Palleschi C. Barra D. Mangoni M. L. (2010). Anti-*Pseudomonas* activity of frog skin antimicrobial peptides in a *Caenorhabditis elegans* infection model: a plausible mode of action in vitro and in vivo. *Antimicrob. Agents Chemother.* 54 3853–3860. 10.1128/AAC.00154-10 20606068 PMC2935021

[B34] Vaillancourt K. LeMay J. D. Lamoureux M. Frenette M. Moineau S. Vadeboncoeur C. (2004). Characterization of a galactokinase-positive recombinant strain of *Streptococcus thermophilus*. *Appl. Environ. Microbiol.* 70 4596–4603. 10.1128/AEM.70.8.4596-4603.2004 15294791 PMC492372

[B35] Virk B. Correia G. Dixon D. P. Feyst I. Jia J. Oberleitner N. (2012). Excessive folate synthesis limits lifespan in the *C. elegans: E. coli* aging model. *BMC Biol.* 10:67 10.1186/1741-7007-10-67 PMC3583181 22849329

[B36] Virk B. Jia J. Maynard C. A. Raimundo A. Lefebvre J. Richards S. A. (2016). Folate Acts in E. *coli* to Accelerate *C. elegans* Aging Independently of Bacterial Biosynthesis. *Cell Rep.* 14 1611–1620. 10.1016/j.celrep.2016.01.051 26876180 PMC4767678

[B37] Vizcaíno J. A. Csordas A. del-Toro N. Dianes J. A. Griss J. Lavidas I. (2016). 2016 update of the PRIDE database and related tools. *Nucleic Acids Res.* 44 D447–D456. 10.1093/nar/gkv1145 26527722 PMC4702828

[B38] Weiss N. Schillinger U. Kandler O. (1983). *Lactobacillus lactis*, *Lactobacillus leichmannii* and *Lactobacillus bulgaricus*, subjective synonyms of *Lactobacillus delbrueckii*, and description of *Lactobacillus delbrueckii* subsp. lactis comb. nov. and *Lactobacillus delbrueckii* subsp. bulgaricus comb. nov. *Syst. Appl. Microbiol.* 4 552–557. 10.1016/S0723-2020(83)80012-5 23194812

[B39] Yilmaz L. S. Walhout A. J. (2014). Worms, bacteria, and micronutrients: an elegant model of our diet. *Trends Genet.* 30 496–503. 10.1016/j.tig.2014.07.010 25172020 PMC4399232

[B40] Yu L. Yan X. Ye C. Zhao H. Chen X. Hu F. (2015). Bacterial respiration and growth rates affect the feeding preferences, brood size and lifespan of *Caenorhabditis elegans*. *PLoS ONE* 10:e0134401 10.1371/journal.pone.0134401 PMC4519269 26222828

[B41] Yun H. S. Heo J. H. Son S. J. Park M. R. Oh S. Song M. H. (2014). *Bacillus licheniformis* isolated from Korean traditional food sources enhances the resistance of *Caenorhabditis elegans* to infection by *Staphylococcus aureus*. *J. Microbiol. Biotechnol.* 24 1105–1108. 10.4014/jmb.1406.06008 24912555

[B42] Zanni E. Laudenzi C. Schifano E. Palleschi C. Perozzi G. Uccelletti D. (2015). Impact of a complex food microbiota on energy metabolism in the model organism *Caenorhabditis elegans*. *Biomed. Res. Int.* 2015:621709 10.1155/2015/621709 PMC4417589 25961031

[B43] Zhao Y. Karypis G. (2005). Data clustering in life sciences. *Mol. Biotechnol.* 31 55–80. 10.1385/MB:31:1:055 16118415

[B44] Zhao Y. Yu X. Jia R. Yang R. Rui Q. Wang D. (2015). Lactic acid bacteria protects *Caenorhabditis elegans* from toxicity of graphene oxide by maintaining normal intestinal permeability under different genetic backgrounds. *Sci. Rep.* 5:17233 10.1038/srep17233 PMC4661518 26611622

