# Supplementary material for: Combination of Metabolomic and Proteomic Analysis Revealed Different Features among Lactobacillus delbrueckii Subspecies bulgaricus and lactis Strains While In Vivo Testing in the Model Organism Caenorhabditis elegans Highlighted Probiotic Properties
Source: Front Microbiol. 2017 Jun 28;8:1206. doi: 10.3389/fmicb.2017.01206 (PMC5487477; doi:10.3389/fmicb.2017.01206)
Supplement: Supplementary file 3 [file Table_3.DOCX]

| **Metabolite** | **Structure** | **Assignment** | **^1^H (ppm)** | **Multiplicity** |
| --- | --- | --- | --- | --- |
| Glutamic acid | 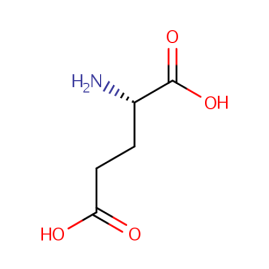 | α-CH  γ-CH_2_  β-CH_2_ | 3.76  2.36  2.09 | m  m |
| Alanina | 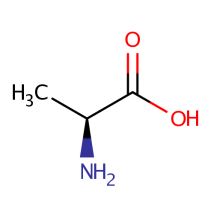 | α-CH  β-CH_3_ | 3.78  1.48 | d |
| Arginine | 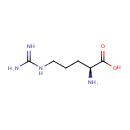 | α-CH  δ-CH_2_  β-CH_2_  γ-CH_2_ | 3.78  3.25  1.90  1.72 |  |
| Asparagine | 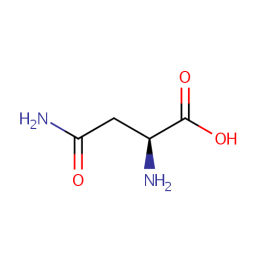 | α-CH  β-CH_2_ | 4.02  2.91 | m |
| Phenylalanine | 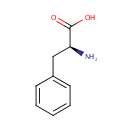 | CH-2,3,4,5,6ring | 7.21-7.43 | m |
| Glutamine | 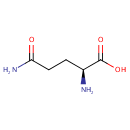 | α-CH  γ-CH_2_  β-CH_2_ | 3.78  2.45  2.15 | m |
| Isoleucine | 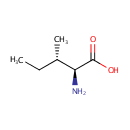 | α-CH  β-CH  γ-CH_3_ | 3.66  1.98  1.01 | d |
| Leucine | 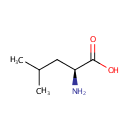 | α-CH  β-CH_2_ γ-CH  ε,ε’-CH_3_ | 3.72  1.73  0.96 | m  m  t |
| Lysine | 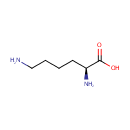 | α-CH  ε-CH_2_  β-CH_2_  δ-CH_2_  γ-CH_2_ | 3.72  3.02  1.90  1.73  1.43 | m |
| Methionine | 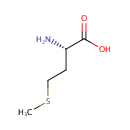 | α-CH  γ-CH_2_  β-CH_2_, ε-CH_3_ | 3.87  2.64  2.15 | t |
| Tyrosine | 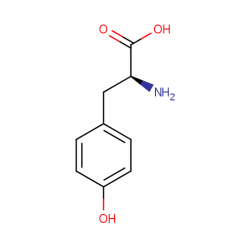 | CH-2,6 ring  CH-3,5 ring | 7.18  6.85 | m  m |
| Threonine | 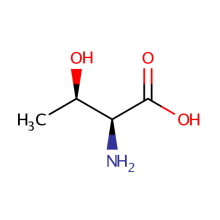 | β-CH  α-CH  γ-CH_3_ | 4.32  3.66  1.32 | q |
| Valine | 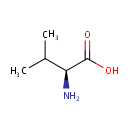 | α-CH  β-CH  γ-CH_3_  γ’-CH_3_ | 3.60  2.26  1.04  0.98 | d  m  d  d |
| 4-hydroxy-phenylacetate | 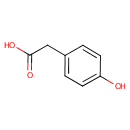 | CH-2,6 ring  CH-3,5 ring | 7.12  6.85 | d |
| Acetate | 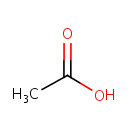 | CH_3_-2 | 1.94 | s |
| Citrate | 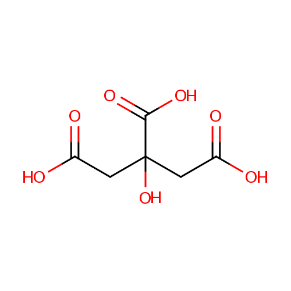 | CH-1,3  CH-1’,3’ | 2.72  2.54 | d  d |
| Formate | 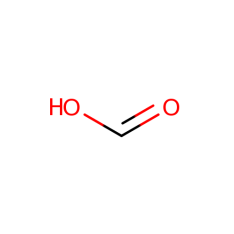 | CH | 8.45 | s |
| Lactate | 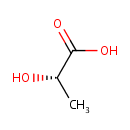 | CH-2  CH_3_-3 | 4.14  1.34 | q  d |
| Pyruvate | 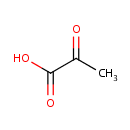 | CH_3_-3 | 2.37 | s |
| Succinate | 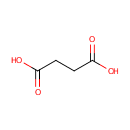 | CH_2_-2,3 | 2.47 | s |
| Methanol | 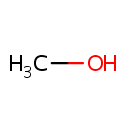 | CH_3_ | 3.35 | s |
| Galactose | 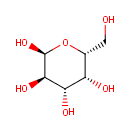 | α-CH-1  β-CH-1 | 5.28  4.62 | d  d |
| Glucose | 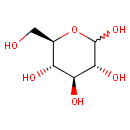 | α-CH-1  β-CH-1 | 5.26  4.58 | d  d |
| Lactose | 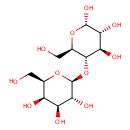 | αGlc-CH-1  βGlc-CH-1  Gal(β1-4) | 5.23  4.67  4.46 | d  d  d |
| Adenosine | 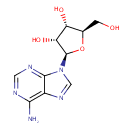 | CH-2  CH-1’  CH-2’  CH-3’ | 8.27  6.02  4.82  4.37 | s  d  s |
| Cytosine | 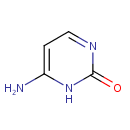 | CH-6  CH-5 | 7.89  5.91 | d  d |
| Guanine | 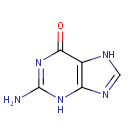 | CH-8 | 7.68 | s |
| Uracil | 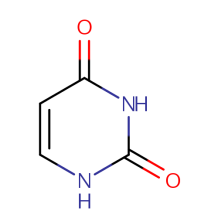 | CH-6  CH-5 | 7.53  5.80 | d  d |
| Choline | 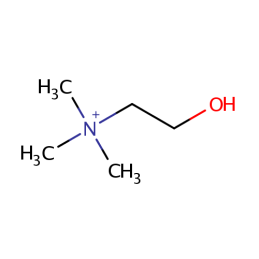 | N(CH_3_)_3_ | 3.19 | s |
| Tyramine | 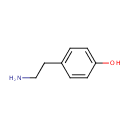 | CH-2,6 ring  CH-3,5 ring | 7.20  6.90 | m  m |
| Saturated fatty acid |  | CH_3_  n-CH_2_  CH_2_-CH_2_-CO_2_^-^  CH_2_-CO_2_^-^ | 0.88  1.27  1.62  2.31 | t  m  m  t |
| Mono-unsaturated fatty acid |  | CH_3_  n-CH_2_  CH_2_-CH=CH  CH=CH  CH_2_-CH_2_-CO_2_^-^  CH_2_-CO_2_^-^ | 0.88  1.27  2.03  5.35  1.62  2.31 | t  m  m  m  m  t |

**Table S3**. NMR signal assignment of metabolites measured in cell extracts of *Lactobacillus delbrueckii* subspecies.

| **Metabolite** | **Structure** | **Assignment** | **^1^H (ppm)** | **Multiplicity** |
| --- | --- | --- | --- | --- |
| Glutamic acid | 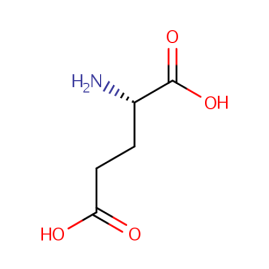 | α-CH  γ-CH_2_  β-CH_2_ | 3,76  2,36  2,09 | m  m |
| Alanina | 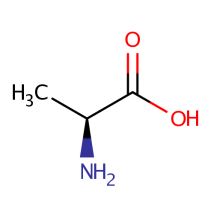 | α-CH  β-CH_3_ | 3,78  1,48 | d |
| Arginine | 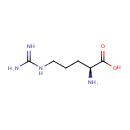 | α-CH  δ-CH_2_  β-CH_2_  γ-CH_2_ | 3,78  3,25  1,90  1,72 |  |
| Asparagine | 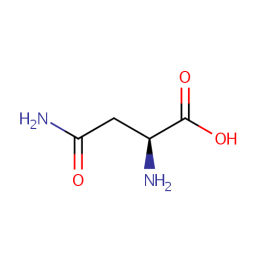 | α-CH  β-CH_2_ | 4,02  2,91 | m |
| Phenylalanine | 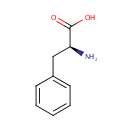 | CH-2,3,4,5,6ring | 7,21-7,43 | m |
| Glutamine | 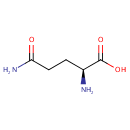 | α-CH  γ-CH_2_  β-CH_2_ | 3,78  2,45  2,15 | m |
| Isoleucine | 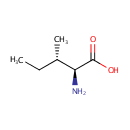 | α-CH  β-CH  γ-CH_3_ | 3,66  1,98  1,01 | d |
| Leucine | 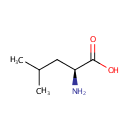 | α-CH  β-CH_2_ γ-CH  ε,ε’-CH_3_ | 3,72  1,73  0,96 | m  m  t |
| Lysine | 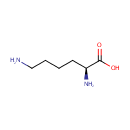 | α-CH  ε-CH_2_  β-CH_2_  δ-CH_2_  γ-CH_2_ | 3,72  3,02  1,90  1,73  1,43 | m |
| Methionine | 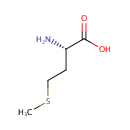 | α-CH  γ-CH_2_  β-CH_2_, ε-CH_3_ | 3,87  2,64  2,15 | t |
| Tyrosine | 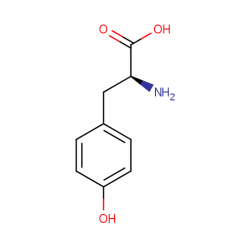 | CH-2,6 ring  CH-3,5 ring | 7,18  6,85 | m  m |
| Threonine | 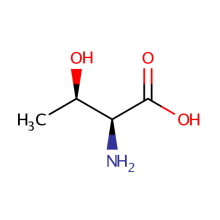 | β-CH  α-CH  γ-CH_3_ | 4,32  3,66  1,32 | q |
| Valine | 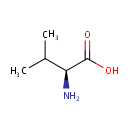 | α-CH  β-CH  γ-CH_3_  γ’-CH_3_ | 3,60  2,26  1,04  0,98 | d  m  d  d |
| 4-hydroxy-phenylacetate | 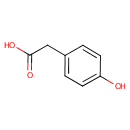 | CH-2,6 ring  CH-3,5 ring | 7,12  6,85 | d |
| Acetate | 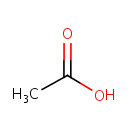 | CH_3_-2 | 1,94 | s |
| Citrate | 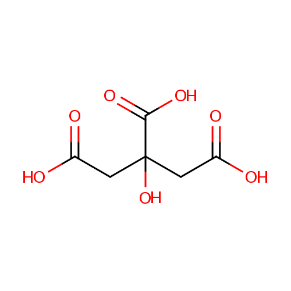 | CH-1,3  CH-1’,3’ | 2,72  2,54 | d  d |
| Formate | 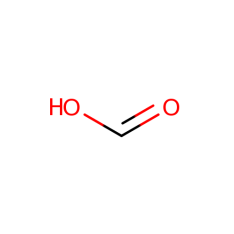 | CH | 8,45 | s |
| Lactate | 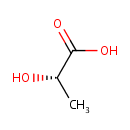 | CH-2  CH_3_-3 | 4,14  1,34 | q  d |
| Pyruvate | 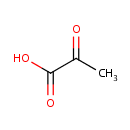 | CH_3_-3 | 2,37 | s |
| Succinate | 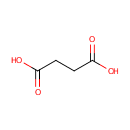 | CH_2_-2,3 | 2,47 | s |
| Methanol | 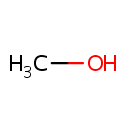 | CH_3_ | 3,35 | s |
| Galactose | 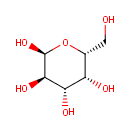 | α-CH-1  β-CH-1 | 5,28  4,62 | d  d |
| Glucose | 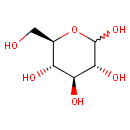 | α-CH-1  β-CH-1 | 5,26  4,58 | d  d |
| Lactose | 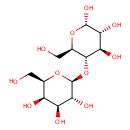 | αGlc-CH-1  βGlc-CH-1  Gal(β1-4) | 5,23  4,67  4,46 | d  d  d |
| Adenosine | 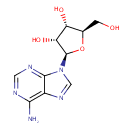 | CH-2  CH-1’  CH-2’  CH-3’ | 8,27  6,02  4,82  4,37 | s  d  s |
| Cytosine | 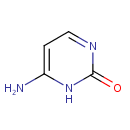 | CH-6  CH-5 | 7,89  5,91 | d  d |
| Guanine | 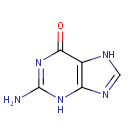 | CH-8 | 7,68 | s |
| Uracil | 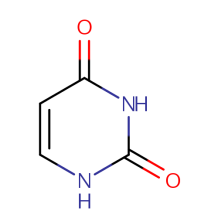 | CH-6  CH-5 | 7,53  5,80 | d  d |
| Choline | 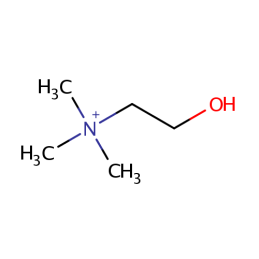 | N(CH_3_)_3_ | 3,19 | s |
| Tyramine | 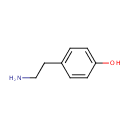 | CH-2,6 ring  CH-3,5 ring | 7,20  6,90 | m  m |
| Saturated fatty acid |  | CH_3_  n-CH_2_  CH_2_-CH_2_-CO_2_^-^  CH_2_-CO_2_^-^ | 0.88  1.27  1.62  2.31 | t  m  m  t |
| Mono-unsaturated fatty acid |  | CH_3_  n-CH_2_  CH_2_-CH=CH  CH=CH  CH_2_-CH_2_-CO_2_^-^  CH_2_-CO_2_^-^ | 0.88  1.27  2.03  5.35  1.62  2.31 | t  m  m  m  m  t |
