# Supplementary material for: Combination of Metabolomic and Proteomic Analysis Revealed Different Features among Lactobacillus delbrueckii Subspecies bulgaricus and lactis Strains While In Vivo Testing in the Model Organism Caenorhabditis elegans Highlighted Probiotic Properties
Source: Front Microbiol. 2017 Jun 28;8:1206. doi: 10.3389/fmicb.2017.01206 (PMC5487477; doi:10.3389/fmicb.2017.01206)
Supplement: Supplementary file 4 [file Presentation_1.PPTX]

## Slide 1
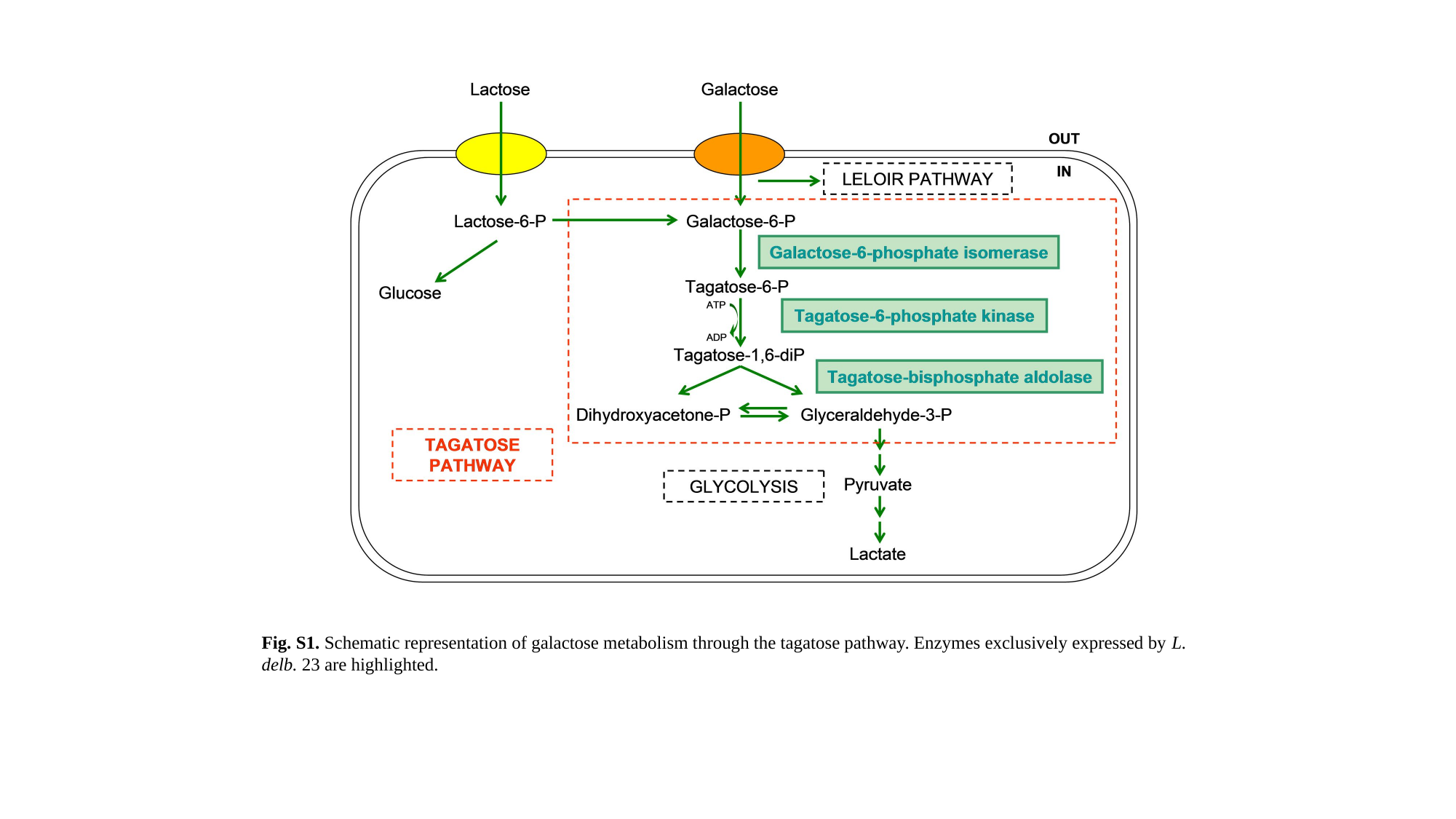

Fig. S1. Schematic representation of galactose metabolism through the tagatose pathway. Enzymes exclusively expressed by L. delb. 23 are highlighted.
