# Supplementary material for: Combination of Metabolomic and Proteomic Analysis Revealed Different Features among Lactobacillus delbrueckii Subspecies bulgaricus and lactis Strains While In Vivo Testing in the Model Organism Caenorhabditis elegans Highlighted Probiotic Properties
Source: Front Microbiol. 2017 Jun 28;8:1206. doi: 10.3389/fmicb.2017.01206 (PMC5487477; doi:10.3389/fmicb.2017.01206)
Supplement: Supplementary file 5 [file Presentation_2.PPTX]

## Slide 1
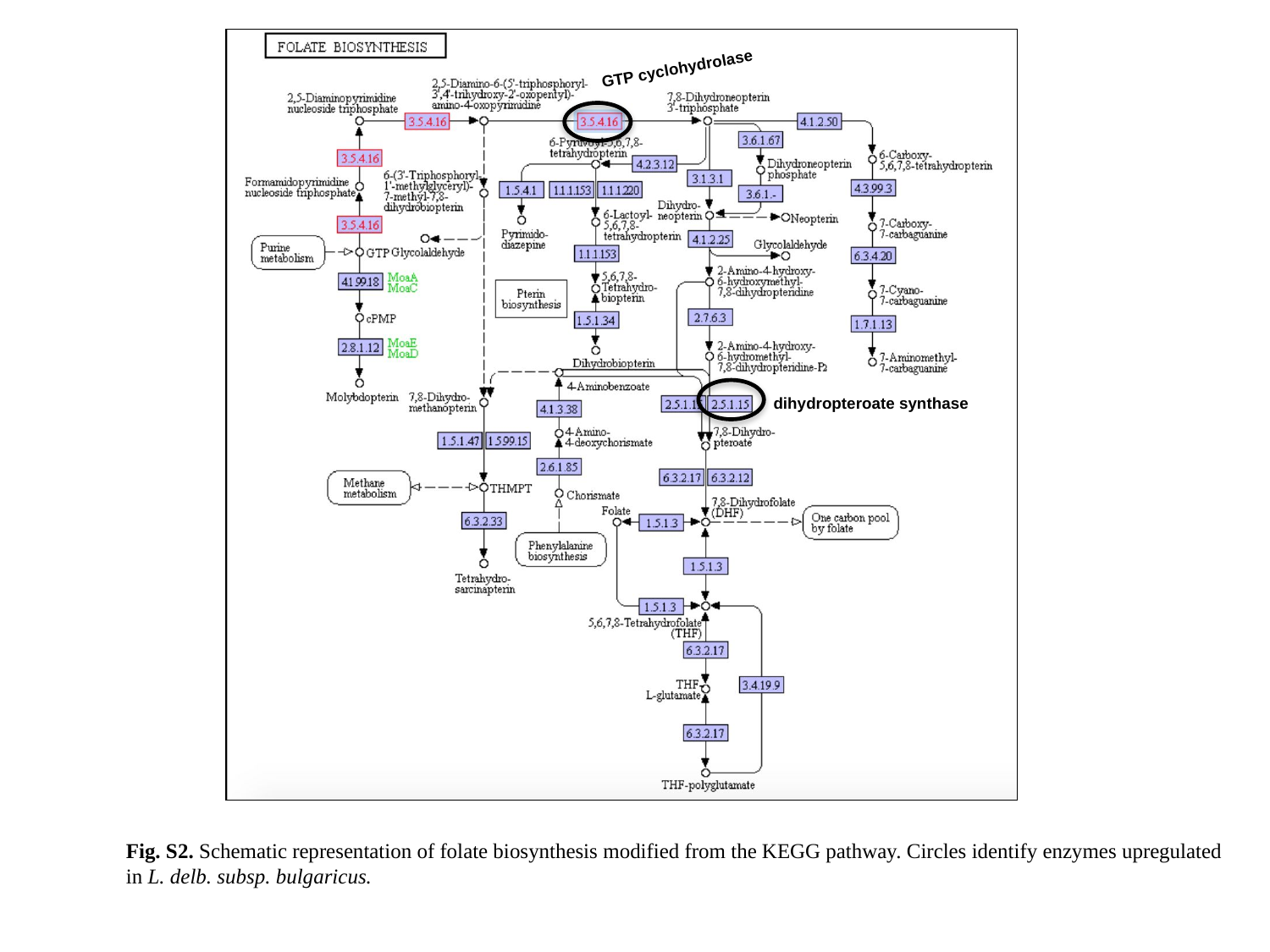

GTP cyclohydrolase
dihydropteroate synthase
Fig. S2. Schematic representation of folate biosynthesis modified from the KEGG pathway. Circles identify enzymes upregulated in L. delb. subsp. bulgaricus.
