# Supplementary material for: Combination of Metabolomic and Proteomic Analysis Revealed Different Features among Lactobacillus delbrueckii Subspecies bulgaricus and lactis Strains While In Vivo Testing in the Model Organism Caenorhabditis elegans Highlighted Probiotic Properties
Source: Front Microbiol. 2017 Jun 28;8:1206. doi: 10.3389/fmicb.2017.01206 (PMC5487477; doi:10.3389/fmicb.2017.01206)
Supplement: Supplementary file 6 [file Image_3.pdf]

(A)

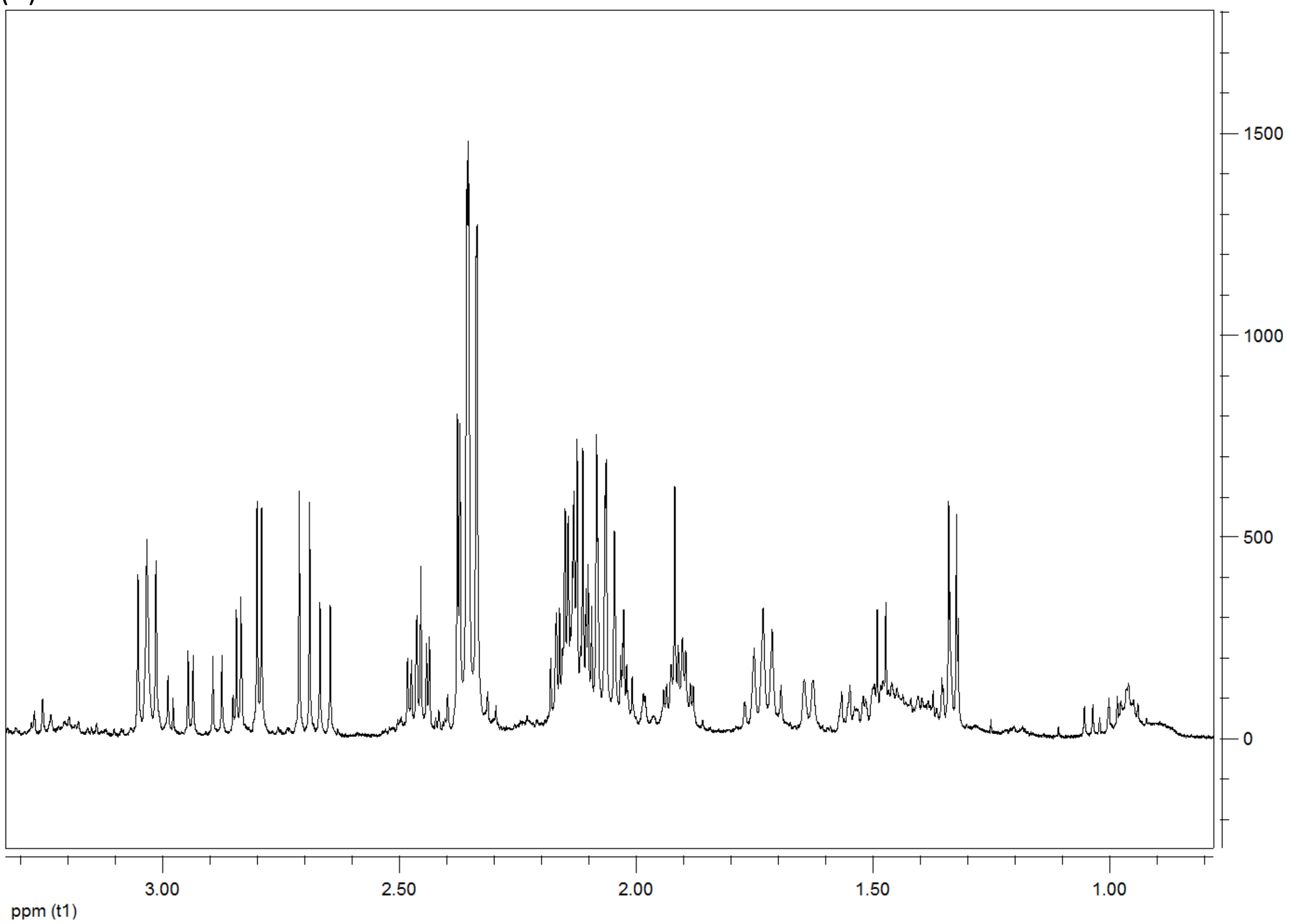

(B)

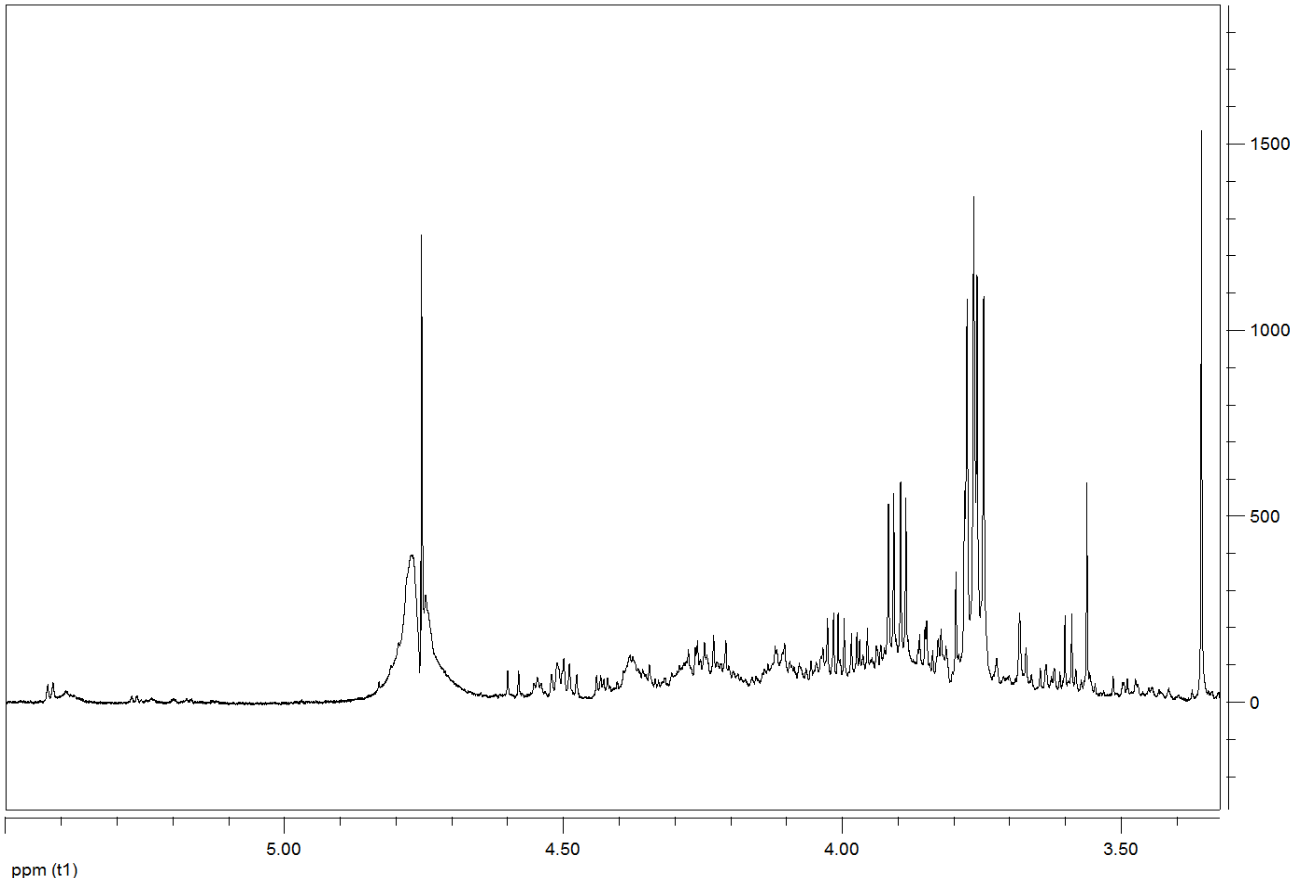

(C)

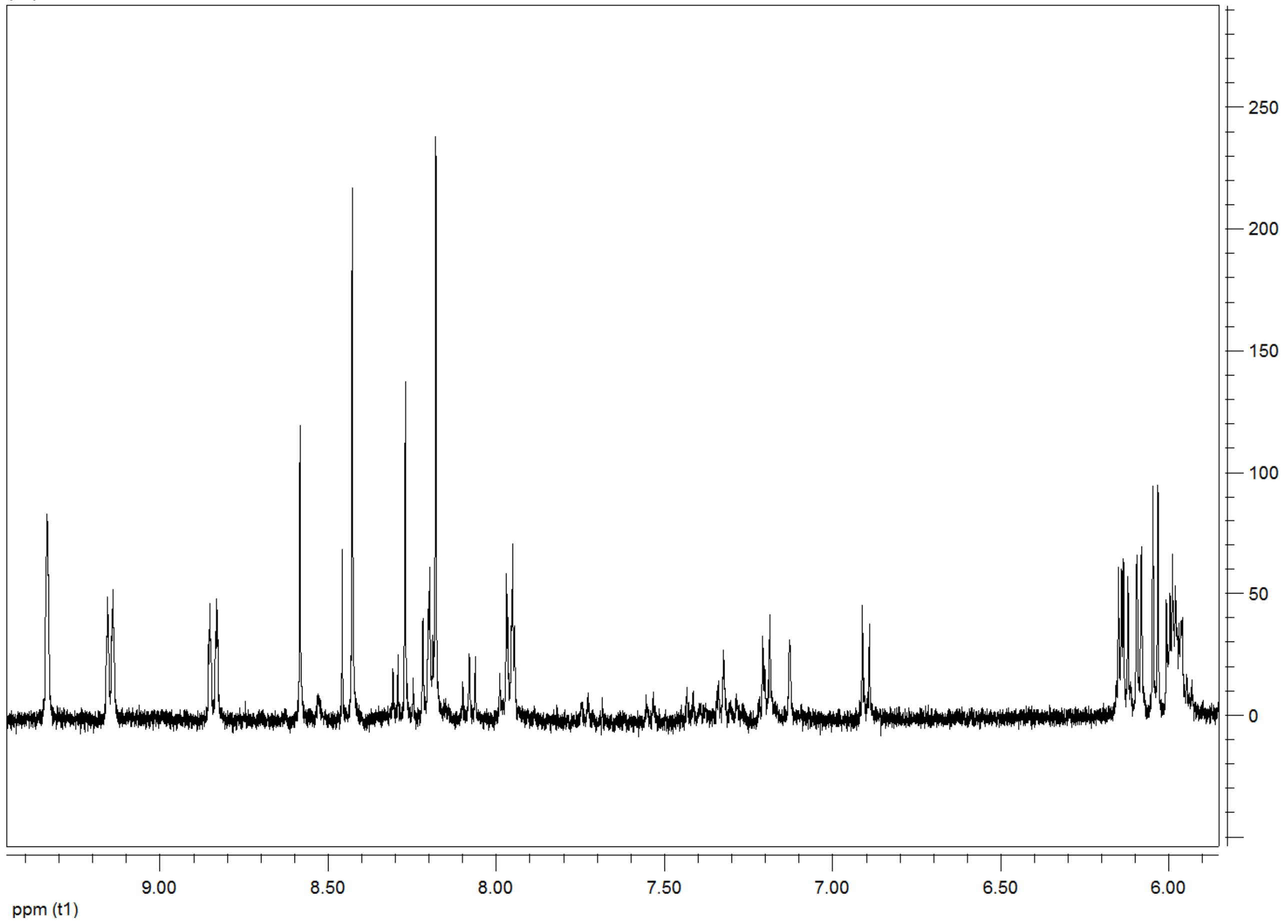

Fig. S3. Typical ^{1}H NMR spectrum of *L. delbrueckii* 23 cell extracts. Spectral region: (A) from 0.8 to 3.3 ppm, (B) from 3.3 ppm to 6.0 ppm and (C) from 6.0 ppm to 9.5 ppm (the intensity of the spectrum has been increased x 4).
